# Correlation between detergent activity and anti-herpes simplex virus-2 activity of commercially available vaginal gels

**DOI:** 10.1186/s13104-020-4918-4

**Published:** 2020-01-31

**Authors:** Andrea Szöllősi, Tímea Raffai, Anita Bogdanov, Valéria Endrész, László Párducz, Ferenc Somogyvári, László Janovák, Katalin Burián, Dezső P. Virok

**Affiliations:** 1 Department of Health and Social Sciences, Gál Ferenc College, Szent István st. 17-19, Gyula, 5700 Hungary; 2 Department of Medical Microbiology and Immunobiology, University of Szeged, Dóm sqr. 10, Szeged, 6720 Hungary; 3 Pándy Kálmán County Hospital, Semmelweis st. 1, Gyula, 5700 Hungary; 4 Interdisciplinary Excellence Centre, Department of Physical Chemistry and Materials Science, University of Szeged, Rerrich Béla sqr. 1, Szeged, 6720 Hungary

**Keywords:** Herpes, Simplex, HSV, Replication, Transmission, Gel, qPCR, Vaginal, STD

## Abstract

**Objective:**

Herpes simplex virus-2 (HSV-2) infections are almost exclusively sexually transmitted. The presence of vaginal gels during sexual activity may have a significant positive or negative impact on viral transmission. Therefore we investigated three off-the-shelf vaginal lubricants and one pH restoring gel to evaluate their impact on HSV-2 replication.

**Results:**

HeLa cells were infected with untreated virions and virions incubated with the particular gels. The accumulation of viral genomes was monitored by quantitative PCR (qPCR) method at 24 h post infection. Two of the tested gels had no significant effect on HSV-2 replication at the maximum applied concentration, while two had a strong inhibitory effect (~ 98% reduction of replication). The replication inhibitory effect was observed at various multiplicity of infection (MOI 0.4–6.4) and the two inhibitory gels were also capable of inhibiting the HSV-2 induced cytopathic effect on HeLa cells. The surface tension decreasing activity—an indication of detergent activity—was strongly correlated with the anti-HSV-2 activity of the gels (R^2^: 0.88). Our results indicate that off-the-shelf vaginal gels have a markedly different anti-HSV-2 activity that may influence HSV-2 transmission.

## Introduction

HSV-2, a member of the family *Herpesviridae*, is an enveloped DNA virus. Herpes simplex virus infections spread through direct contact with body fluids, and in the case of HSV-2 the transmission is principally sexual. The seroprevalence of HSV-2 indicates that a significant part of the population harbors the virus [1]. Herpes genitalis, the primary clinical manifestation of HSV-2 infection is local and mainly includes vesicles and ulcers. Genital herpes can lead to significant clinical complications such as neonatal herpesvirus encephalitis [2] and an increase in risk of HIV transmission [3]. Similarly to other herpesviruses, HSV-2 persistence is common and periodic reactivation with and without clinical symptoms is frequent [4]. During reactivation, HSV-2 can be found in vaginal lesions and secretions and can be transmitted. The cervicovaginal microenvironment can profoundly influence HSV-2 transmission. Previous in vitro and epidemiology studies showed that the presence of various *Lactobacillus* species could inhibit HSV-2 development and reduce the prevalence of HSV-2 [5–8]. Chemical compounds such as vaginal gels applied during or before sexual intercourse could also influence the effectivity of HSV-2 transmission. Incorporating microbicides into vaginal gels is a well-accepted strategy for inhibiting sexually transmitted disease transmission including HSV-2 transmission [9–11]. We also showed that even basic components of the vaginal gels, such as the gelling agent hydroxyethyl cellulose can also significantly influence the replication of sexually transmitted pathogens such as *Chlamydia trachomatis* [12].

We recently developed a direct qPCR method with which we can accurately measure herpesvirus genome accumulation in the infected cells in vitro and the replication inhibitory effects of antiviral drugs and neutralizing antibodies [13]. We applied this technology to investigate the effect of off-the-shelf vaginal lubricants and a pH restoring gel on the effectivity of HSV-2 infection of HeLa cervical epithelial cells.

## Main text

### Materials and methods

#### Characterization of the maximum non-toxic concentrations of the applied vaginal gels

3-(4,5-Dimethylthiazol-2-yl)-2,5-diphenyltetrazolium bromide (MTT) assay was performed to calculate the maximum non-toxic concentration of the tested four vaginal gels (lubricants: Gel-1, Gel-2, Gel-4; pH restoring gel: Gel-3). The minimum essential medium (MEM) with Earle’s salts completed with 10% fetal bovine serum (FBS), 2 mmol/L l-glutamine, 1 × nonessential amino acids, 25 μg/mL gentamicin and 0.5 μg/mL fungizone on HeLa cells was complemented with serial twofold dilutions of the vaginal gels for each concentration (n = 3). The initial concentrations of the vaginal gels were 20 w/v% and further dilutions were performed in MEM. After a 24-h incubation, an MTT assay was performed as described earlier [14]. All reagents were purchased from SIGMA (St. Louis, MO, USA), if otherwise not indicated.

#### Assessment of the impact of vaginal gels on HSV-2 replication by direct qPCR

A clinical HSV-2 strain isolated in the Department of Medical Microbiology (University of Szeged, Szeged, Hungary) was used [13, 15]. HeLa cells (6 × 10^4^ cells/well) were seeded into 96-well plates in 100 µL MEM. Next day the HeLa cells were infected with HSV-2 (MOI 0.1) preincubated with a vaginal gel for 1 h, at 37 °C. After the infection (1 h, 37 °C, 5% CO_2_), the inoculum was removed and MEM, 10% FBS medium was added. Each gel concentration was tested in three parallel wells. 24-h post infection, the cells were washed twice with phosphate buffered saline (PBS) and were subjected to two freeze–thaw cycles in 100 μL Milli-Q water to extract the viral DNA. 1 μL of the cell lysates were used as templates in a direct qPCR as described previously [13]. Statistical comparisons of treated samples vs untreated controls (cycle threshold (Ct) values) were performed by Student’s t-test as described previously [16].

#### Measurement of the impact of vaginal gels on the surface tension

The surface tension measurements of diluted gel solutions were performed on a K100 MK2 Tensiometer (Krüss Co., Hamburg, Germany) using the Wilhelmy plate method. The initial concentration of the gel aqueous dilutions was 1.5 g/L for each samples. The surface tension was measured at different concentrations by placing a 40 mL volume of sample solution in sample receptacle and diluting it with deionized water from a connected Dosimat 765 (Metrohm, Herisau, Switzerland) titration stand. The solutions were immersed in a constant temperature bath at the desired temperature (25 ± 0.02 °C). During the automatized surface tension measurements the tensiometer and the dosing unit was controlled using the modularly constructed LabDesk™ software.

### Results

#### Impact of vaginal gels on the viability of HeLa cells

In order to exclude the potential HSV-2 replication inhibitory effects of the vaginal gels due to the inhibition of the host cell metabolism, we measured HeLa cell viability after 24 h of incubation (Additional file 1: Figure S1). Except for Gel-3, cytotoxicity was not observed even at the maximal applied concentration of 20 w/v%. Interestingly, for Gel-1 we were even able to detect a moderate increase of cell viability at the highest concentration. We treated the 20 w/v% (Gel-1, Gel-2, Gel-4) and 10 w/v% concentration (Gel-3) as the maximum non-toxic concentrations, and used them as the first concentrations for the 1:2 dilution series in subsequent experiments.

#### Direct qPCR measurement of the inhibition of HSV-2 replication by antiviral compounds

**Fig. 1:**
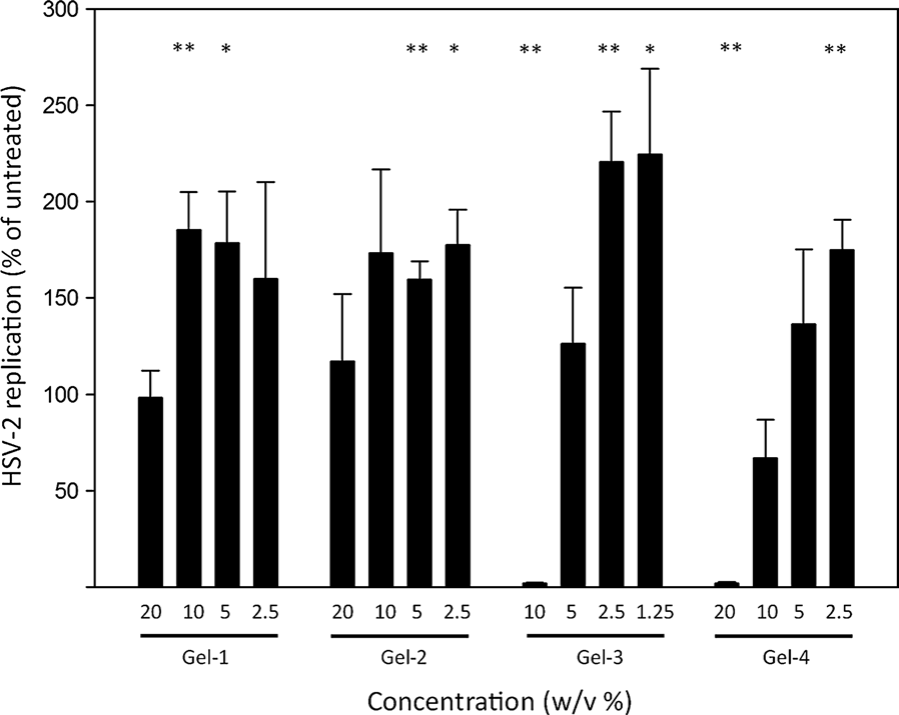
Assessment of the impact of vaginal gels on HSV-2 replication. HeLa cells were infected with HSV-2 preincubated (1 h, 37 ℃) with 20–2.5 w/v% concentrations of Gel-1, Gel-2, Gel-4 gels and 10–1.25 w/v% concentrations of Gel-3. At 24 h post infection, the cells were lysed and the HSV-2 DNA concentration was measured by direct qPCR. Statistical comparison of HSV-2 replication (Ct values of treated samples vs untreated controls (n = 3)) was performed by Student’s t-test. *P < 0.05, **P < 0.01

**Fig. 2:**
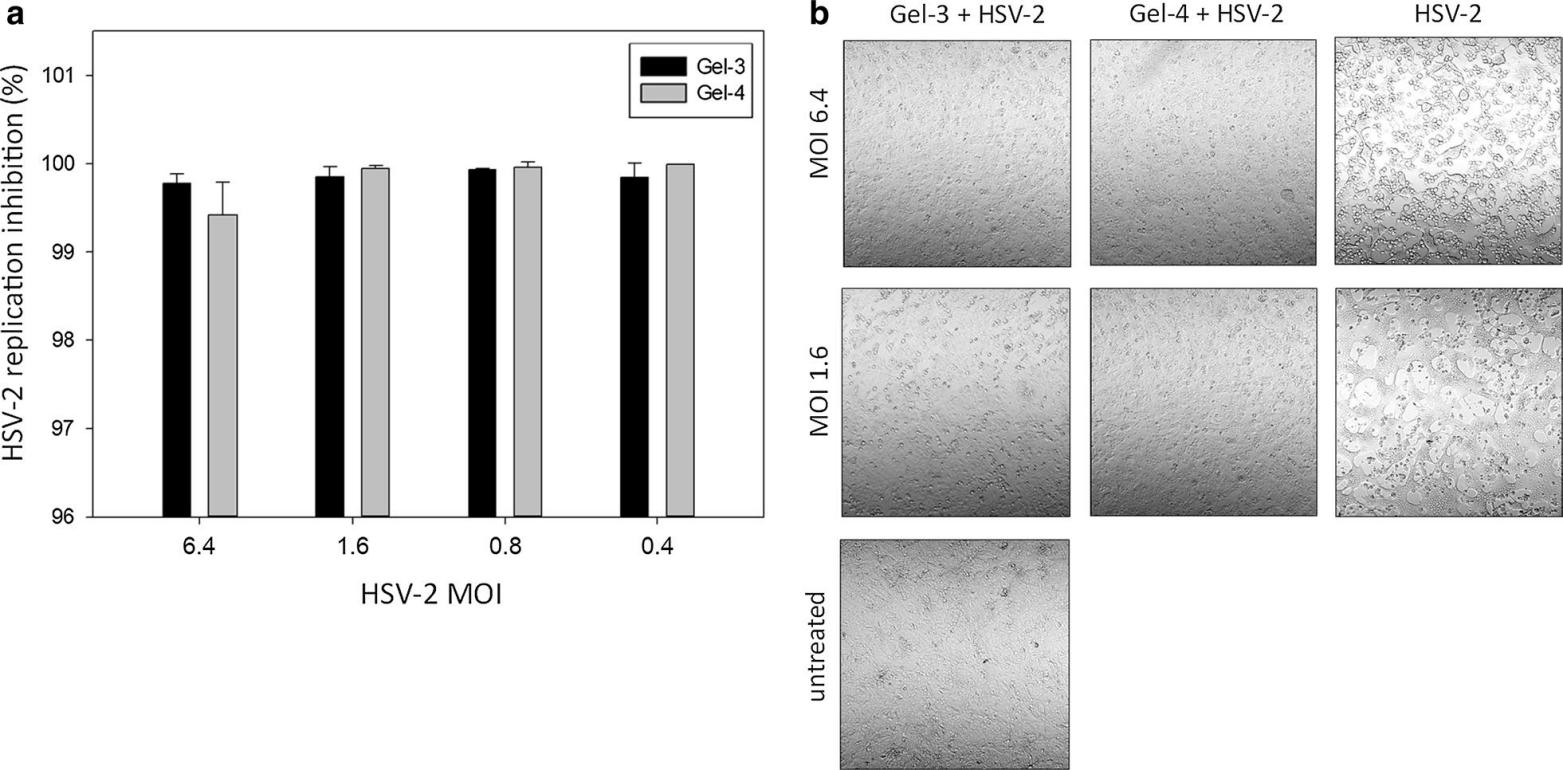
Evaluation of the impact of Gel-3 and Gel-4 on HSV-2 replication and the inhibition of the HSV-2 induced cytopathic effect. **a** HeLa cells were infected with HSV-2 (MOI 6.4–0.4) preincubated (1 h, 37 ℃) with 10 w/v% and 20 w/v% concentrations of Gel-3 and Gel-4, respectively. At 24 h post infection, the cells were lysed and the HSV-2 DNA concentration was measured by direct qPCR (n = 3). **b** HeLa cells were infected with untreated and Gel-3 and Gel-4 treated HSV-2 (MOI 6.4 and MOI 1.6), as described before. HSV-2 cytopathic effect was compared to the untreated HeLa cells by light microscopy 24 h post infection

We applied our recently developed direct qPCR method [13] to assess the impact of vaginal gels on HSV-2 replication. We infected HeLa cells with HSV-2 in the presence of serial dilutions of the vaginal gels, starting with the maximum non-toxic concentrations (Fig. 1). Based on their impact on HSV-2 replication, the four tested gels could be divided into two groups. Gel-1 and Gel-2 were not able to inhibit HSV-2 replication even at the highest applied concentration, while Gel-3 and Gel-4 strongly inhibited HSV-2 replication at the maximum applied concentrations. In the case of Gel-3, the HSV-2 replication inhibition was 98.2%, and for Gel-4 the replication inhibition was 98.1%. Further dilutions of all the four gels behaved similarly: reduced to a lesser amount or slightly increased the replication of HSV-2. To evaluate whether the antiviral activity of Gel-3 and Gel-4 could be detected against different viral loads, we performed experiments with MOIs ranging from 0.4 to 6.4 (Fig. 2a). Similar to the previous experiments, Gel-3 and Gel-4 had a ~ 99% inhibitory effect in the 0.4–6.4 MOI range. In correlation with their significant antiviral activity, Gel-3 and Gel-4 also prevented the cytopathic effect of HSV-2 at MOI 6.4 and MOI 1.6 (Fig. 2b).

#### The impact of vaginal gels on surface tension

**Fig. 3:**
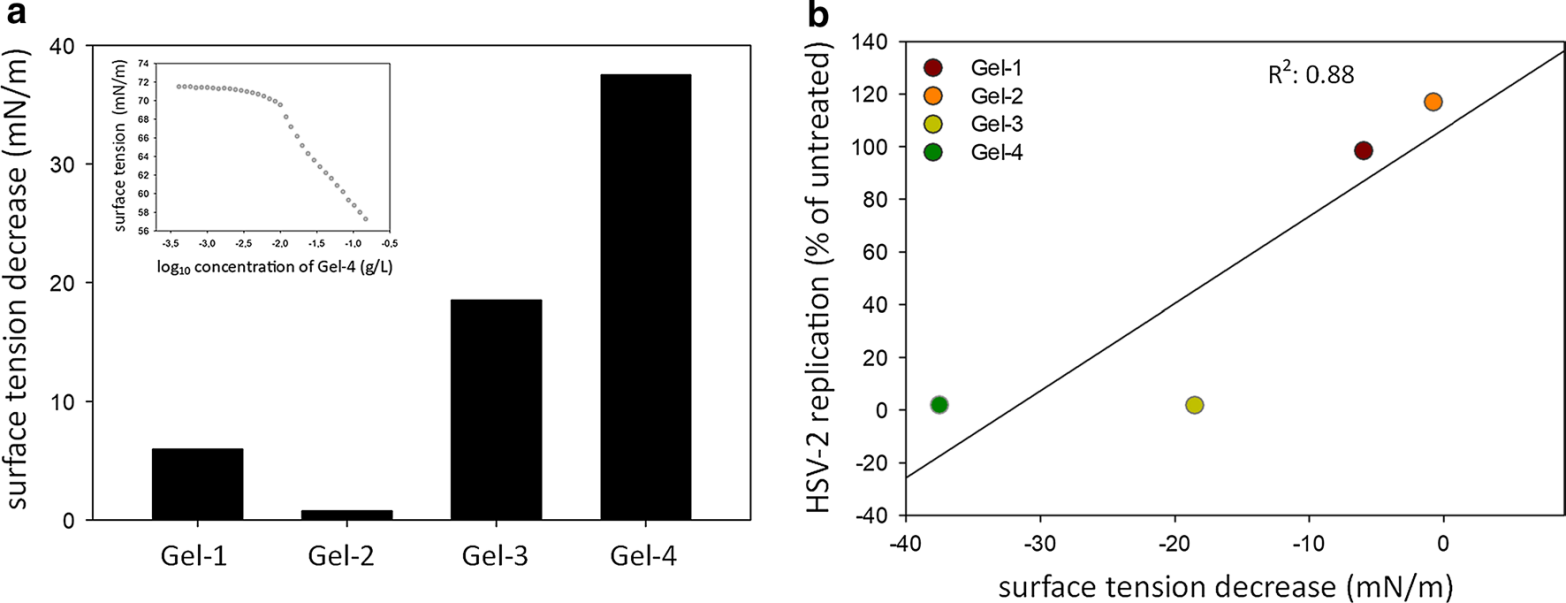
Association between the surface tension decreasing effects and the antiviral effects of the vaginal gels. **a** Surface tension decreasing activity of the tested gels. Surface tension decrease was calculated by subtraction of surface tension in the minimum gel concentration and surface tension in the maximum gel concentration. Insert shows surface tension decrease by Gel-4 as a function of log_10_ gel concentration. **b** Correlation between the surface tension decreasing activity and the average HSV-2 replication measured at the maximum gel concentration. Pearson correlation coefficient is also shown

To assess the potential detergent activity of the vaginal gels, we measured their surface tension decreasing effect. The measured surface tension values of the vaginal gel dilutions were plotted against the logarithm of the total concentration at 25 ± 0.02 °C (Fig. 3a, inset) and the difference between surface tensions measured the minimum and maximum gel concentrations were calculated. Gel-3 and Gel-4 had the highest surface tension decreasing effect with 18 mN/m and 33 mN/m respectively, while Gel-1 and Gel-2 only minimally decreased the surface tension (5.9 mN/m and 0.8 mN/m respectively) (Fig. 3a). The surface tension decreasing effects strongly correlated with the HSV-2 replication inhibitory activity (R^2^ 0.88) (Fig. 3b).

### Discussion

We tested four commercially available vaginal gels to assess their HSV-2 replication modulating activity in vitro. Our data showed that the tested gels either had an approximately neutral effect (Gel-1, Gel-2) or strong inhibitory effect (Gel-3, Gel-4) at the highest tested concentration (10–20 w/v%). Since for Gel-3 and Gel-4, the tested 10–20 w/v% concentrations mean in effect a five-tenfold dilution, and considering that the volume of vaginal fluid and sperm lies in the 1–3 mL range [17, 18], one may expect that in vivo these gels can achieve 10–20 w/v% or higher concentrations and have significant antiviral activity. As during the symptomatic infection the sexual activity is likely abandoned, HSV-2 transmission via shedding during the asymptomatic periods and in the very early phase of symptomatic infections is probably more frequent. Daily testing of HSV-2 seropositive individuals revealed that asymptomatic HSV-2 shedding occurred in 2–3.8% of the days [19, 20], indicating that asymptomatic HSV-2 shedding is a relatively frequent event. Schiffer et al. showed that for 14,685 swab samples, 18% were HSV-2 positive (> 150 DNA copies/ml), and close to 90% of the samples contained more than 10^4^ DNA copies/mL [21]. Since the median HSV-2 load was 10^4.8^ DNA copies/mL [21] and the threshold of HSV-2 transmission was calculated previously as > 10^4^ infectious unit (IFU) [22], even a low level HSV-2 load decrease could be significant in preventing the transmission. Gel-3 and Gel-4 were able to cause ~ 2 logs decrease in HSV-2 IFU and were effective at least at 6.4 MOIs (~ 400,000 IFUs), therefore these gels might lower the risk of transmission, despite the fact that they were not designed for prevention. Altogether, these results highlight the importance of evaluation of commercially available vaginal gels/lubricants for their possible anti-HSV-2 activity before investigating their role in the prevention of HSV-2 transmission [23].

As the commercially available gels generally contain several ingredients in unknown concentrations, the exact sources of the cumulative inhibitory activity are not known. As an example, a potentially antimicrobial unique component of Gel-4 gel was the “citrus aroma”. It was described previously, that citrusinine-I, an alkaloid isolated from the citrus plant Rutaceae displayed antiviral activity against HSV-1 and HSV-2 [24]. Among the physicochemical attributes of the vaginal gels, one is their hydrophilic, hydrophobic or amphipathic nature. Amphipathic gel components can behave as surfactants, providing a detergent-like activity for the gels. HSV-2 is an enveloped virus, hence the detergent activity could destabilize the viral membrane and decrease viral infectivity [25]. We measured the surface tension decreasing activity of the gels which correlates with their detergent-like activity. Gel-3 and Gel-4 showed a marked detergent-like activity, while Gel-1 and Gel-2 had negligible effect. Our results also showed that detergent-like activity and the in vitro antiviral activity of the gels were strongly correlated, a finding that may be used in future gel developments.

### Limitations

A limitation of our study, that we did not investigate the in vivo antiviral effects of the gels. Previous data show that in vivo a marked detergent-like activity may actually increase HSV-2 susceptibility. In a mouse model of HSV-2 infection, all the five intravaginally applied detergent containing gels increased HSV-2 susceptibility [26]. This effect was likely due to the in vivo detergent cytotoxicity, which led to injury and shedding of the epithelial cells from the upper layers of the mucosa. It is possible that there is an optimal detergent concentration where the viral membrane is destabilized, but the epithelial layer of the cervix remains intact. Regarding the selective toxicity, two of the four tested gels showed significant anti-HSV-2 activity but had no impact on the viability of the host cells in the 1:5 and 1:10 dilutions (20 w/v% and 10 w/v%). However there is room for further development, e.g. Gel-3 showed anti-HSV-2 activity only in its first non-toxic concentration.

In conclusion, our experiments revealed that there are substantial differences among commercially available vaginal gels regarding their anti-HSV-2 activity. From the tested four gels we found two with a significant antiviral activity, suggesting that these gels might be able to decrease the frequency of HSV-2 transmission. Further experiments are needed to evaluate their overall effect on HSV-2 infectivity in vivo.

## Supplementary information

**Additional file 1: Figure S1.** MTT cell viability assay of HeLa cells incubated with the vaginal gels. Viability of the gel-treated cells were compared to the untreated controls. Data are mean ± SD (n = 3). Statistical comparisons of cell viabilities (treated vs. untreated control) were performed by Student’s t-test. *: *P *< 0.05.

## Data Availability

Not applicable.

## Abbreviations

HSV-2: Herpes simplex virus-2
qPCR: Quantitative PCR
MOI: Multiplicity of infection
MTT: 3-(4,5-Dimethylthiazol-2-yl)-2,5-diphenyltetrazolium bromide
MEM: Minimum essential medium
FBS: Fetal bovine serum
PBS: Phosphate buffered saline
IFU: Infectious unit

## References

[1] Torrone EA, Morrison CS, Chen P-L, Kwok C, Francis SC, Hayes RJ (2018). Prevalence of sexually transmitted infections and bacterial vaginosis among women in sub-Saharan Africa: an individual participant data meta-analysis of 18 HIV prevention studies. PLoS Med..

[2] Pinninti SG, Kimberlin DW (2018). Neonatal herpes simplex virus infections. Semin Perinatol.

[3] Desai DV, Kulkarni SS (2015). Herpes simplex virus: the interplay between HSV, host, and HIV-1. Viral Immunol.

[4] Agyemang E, Magaret AS, Selke S, Johnston C, Corey L, Wald A (2018). Herpes simplex virus shedding rate: surrogate outcome for genital herpes recurrence frequency and lesion rates, and phase 2 clinical trials end point for evaluating efficacy of antivirals. J Infect Dis.

[5] Mastromarino P, Cacciotti F, Masci A, Mosca L (2011). Antiviral activity of *Lactobacillus brevis* towards herpes simplex virus type 2: role of cell wall associated components. Anaerobe.

[6] Kassaa IA, Hober D, Hamze M, Caloone D, Dewilde A, Chihib N-E (2015). Vaginal *Lactobacillus gasseri* CMUL57 can inhibit herpes simplex type 2 but not Coxsackievirus B4E2. Arch Microbiol.

[7] Mohseni AH, Taghinezhad-S S, Keyvani H, Ghobadi N (2018). Comparison of acyclovir and multistrain *Lactobacillus* brevis in women with recurrent genital herpes infections: a double-blind, randomized, controlled study. Probiotics Antimicrob Proteins..

[8] Borgdorff H, Tsivtsivadze E, Verhelst R, Marzorati M, Jurriaans S, Ndayisaba GF (2014). *Lactobacillus*-dominated cervicovaginal microbiota associated with reduced HIV/STI prevalence and genital HIV viral load in African women. ISME J.

[9] Fields SA, Bhatia G, Fong JM, Liu M, Shankar GN (2015). SR-2P vaginal microbicide gel provides protection against herpes simplex virus 2 when administered as a combined prophylactic and postexposure therapeutic. Antimicrob Agents Chemother.

[10] Villegas G, Calenda G, Zhang S, Mizenina O, Kleinbeck K, Cooney ML (2016). In vitro exposure to PC-1005 and cervicovaginal lavage fluid from women vaginally administered PC-1005 inhibits HIV-1 and HSV-2 infection in human cervical mucosa. Antimicrob Agents Chemother.

[11] Calenda G, Villegas G, Barnable P, Litterst C, Levendosky K, Gettie A (1999). MZC gel inhibits SHIV-RT and HSV-2 in macaque vaginal mucosa and SHIV-RT in rectal mucosa. J Acquir Immune Defic Syndr.

[12] Raffai T, Burián K, Janovák L, Bogdanov A, Hegemann JH, Endrész V (2019). Vaginal gel component hydroxyethyl cellulose significantly enhances the infectivity of *Chlamydia trachomatis* serovars D and E. Antimicrob Agents Chemother.

[13] Virók DP, Eszik I, Mosolygó T, Önder K, Endrész V, Burián K (2017). A direct quantitative PCR-based measurement of herpes simplex virus susceptibility to antiviral drugs and neutralizing antibodies. J Virol Methods.

[14] Mosmann T (1983). Rapid colorimetric assay for cellular growth and survival: application to proliferation and cytotoxicity assays. J Immunol Methods.

[15] Mucsi I, Molnár J, Motohashi N (2001). Combination of benzo[a]phenothiazines with acyclovir against herpes simplex virus. Int J Antimicrob Agents.

[16] Yuan JS, Reed A, Chen F, Stewart CN (2006). Statistical analysis of real-time PCR data. BMC Bioinformatics.

[17] Moncla BJ, Chappell CA, Debo BM, Meyn LA (2016). The Effects of hormones and vaginal microflora on the glycome of the female genital tract: cervical-vaginal fluid. PLoS ONE.

[18] Comar VA, Petersen CG, Mauri AL, Mattila M, Vagnini LD, Renzi A (2017). Influence of the abstinence period on human sperm quality: analysis of 2,458 semen samples. JBRA Assist Reprod..

[19] Wald A, Zeh J, Selke S, Ashley RL, Corey L (1995). Virologic characteristics of subclinical and symptomatic genital herpes infections. N Engl J Med.

[20] Wald A, Zeh J, Selke S, Warren T, Ryncarz AJ, Ashley R (2000). Reactivation of genital herpes simplex virus type 2 infection in asymptomatic seropositive persons. N Engl J Med.

[21] Schiffer JT, Wald A, Selke S, Corey L, Magaret A (2011). The kinetics of mucosal herpes simplex virus-2 infection in humans: evidence for rapid viral-host interactions. J Infect Dis.

[22] Schiffer JT, Mayer BT, Fong Y, Swan DA, Wald A (2014). Herpes simplex virus-2 transmission probability estimates based on quantity of viral shedding. J R Soc Interface.

[23] de Bruyn G, Shiboski S, van der Straten A, Blanchard K, Chipato T, Ramjee G (2011). The effect of the vaginal diaphragm and lubricant gel on acquisition of HSV-2. Sex Transm Infect..

[24] Yamamoto N, Furukawa H, Ito Y, Yoshida S, Maeno K, Nishiyama Y (1989). Anti-herpesvirus activity of citrusinine-I, a new acridone alkaloid, and related compounds. Antiviral Res.

[25] Newcomb WW, Brown JC (2009). Time-dependent transformation of the herpesvirus tegument. J Virol.

[26] Cone RA, Hoen T, Wong X, Abusuwwa R, Anderson DJ, Moench TR (2006). Vaginal microbicides: detecting toxicities in vivo that paradoxically increase pathogen transmission. BMC Infect Dis.

