# Supplementary material for: Correlation between detergent activity and anti-herpes simplex virus-2 activity of commercially available vaginal gels
Source: BMC Res Notes. 2020 Jan 31;13:52. doi: 10.1186/s13104-020-4918-4 (PMC6995179; doi:10.1186/s13104-020-4918-4)
Supplement: Supplementary file 1 — Additional file 1: Figure S1. MTT cell viability assay of HeLa cells incubated with the vaginal gels. Viability of the gel-treated cells were compared to the untreated controls. Data are mean ± SD (n = 3). Statistical comparisons of cell viabilities (treated vs. untreated control) were performed by Student’s t-test. *: P < 0.05. [file 13104_2020_4918_MOESM1_ESM.docx]

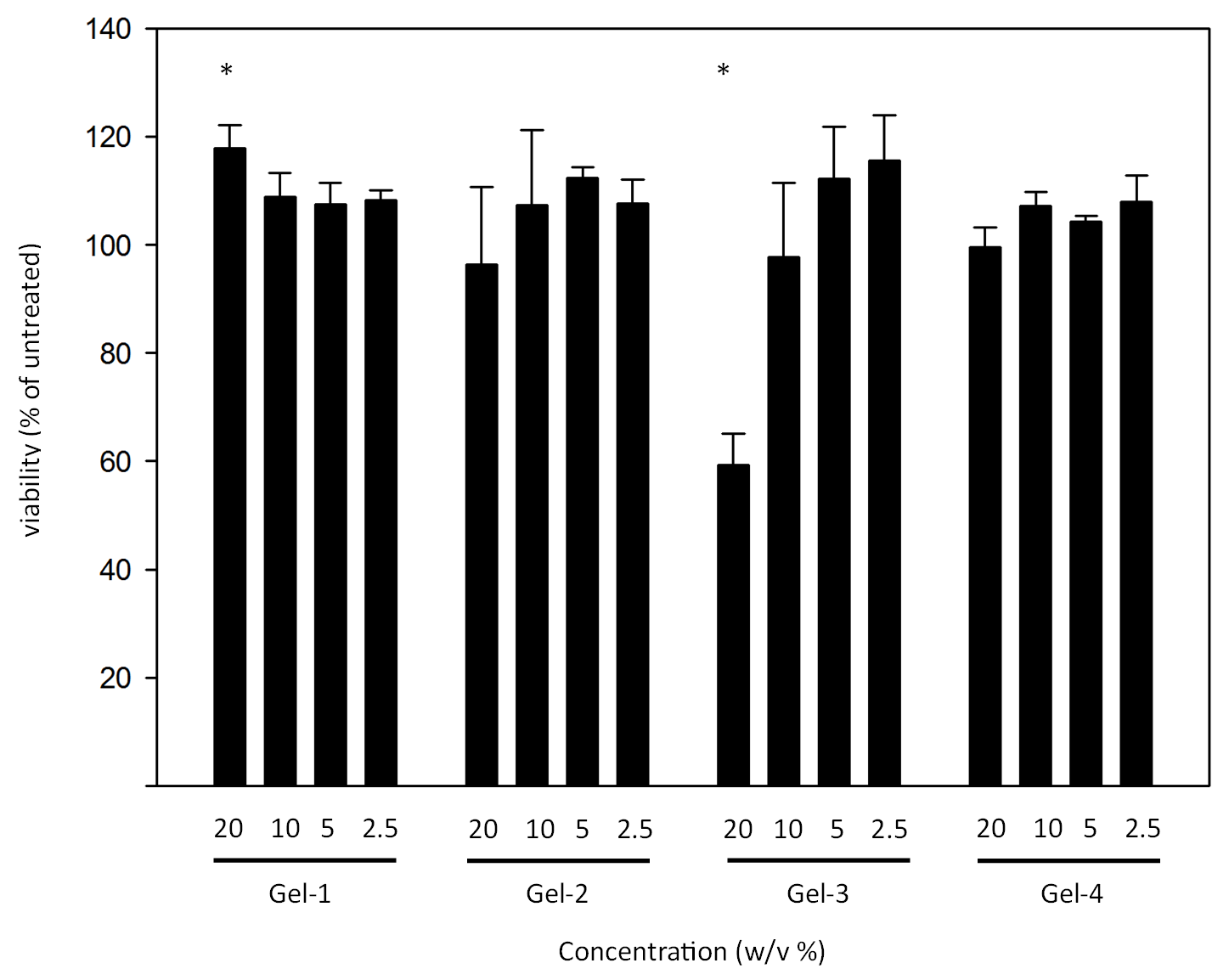


**Supplementary Figure.** MTT cell viability assay of HeLa cells incubated with the vaginal gels. Viability of the gel-treated cells were compared to the untreated controls. Data are mean +/- SD (n=3). Statistical comparisons of cell viabilities (treated vs. untreated control) were performed by Student’s t-test. *: *P*<0.05.
